# Machine Learning-Based Data Mining Method for Sentiment Analysis of the Sewol Ferry Disaster's Effect on Social Stress

**DOI:** 10.3389/fpsyt.2020.505673

**Published:** 2020-12-23

**Authors:** Min-Joon Lee, Tae-Ro Lee, Seo-Joon Lee, Jin-Soo Jang, Eung Ju Kim

**Affiliations:** ^1^BK21PLUS Program in Embodiment: Health-Society Interaction, Department of Health Science, Graduate School, Korea University, Seoul, South Korea; ^2^BK21PLUS Program in Embodiment: Health-Society Interaction, School of Health Policy and Management, Korea University, Seoul, South Korea; ^3^Research Institute of Health Science, Korea University, Seoul, South Korea; ^4^Korea University Research Institute for Medical Bigdata Science, Korea University, Seoul, South Korea; ^5^Division of Cardiology, Department of Medicine, Korea University Guro Hospital, Korea University College of Medicine, Seoul, South Korea

**Keywords:** data mining, sentiment analysis, data crawling, machine learning, natural language processing

## Abstract

The Sewol Ferry Disaster which took place in 16th of April, 2014, was a national level disaster in South Korea that caused severe social distress nation-wide. No research at the domestic level thus far has examined the influence of the disaster on social stress through a sentiment analysis of social media data. Data extracted from YouTube, Twitter, and Facebook were used in this study. The population was users who were randomly selected from the aforementioned social media platforms who had posted texts related to the disaster from April 2014 to March 2015. ANOVA was used for statistical comparison between negative, neutral, and positive sentiments under a 95% confidence level. For NLP-based data mining results, bar graph and word cloud analysis as well as analyses of phrases, entities, and queries were implemented. Research results showed a significantly negative sentiment on all social media platforms. This was mainly related to fundamental agents such as ex-president Park and her related political parties and politicians. YouTube, Twitter, and Facebook results showed negative sentiment in phrases (63.5, 69.4, and 58.9%, respectively), entity (81.1, 69.9, and 76.0%, respectively), and query topic (75.0, 85.4, and 75.0%, respectively). All results were statistically significant (*p* < 0.001). This research provides scientific evidence of the negative psychological impact of the disaster on the Korean population. This study is significant because it is the first research to conduct sentiment analysis of data extracted from the three largest existing social media platforms regarding the issue of the disaster.

## Introduction

On April 16th, 2014, the citizens of South Korea suffered from a high amount of stress due to the Sewol Ferry Disaster, which led to more than 300 casualties (1), most of whom were students. During that time, the disaster was such an important issue that more than 600 journalistic outlets published stories about the subject within less than a week (2).

This sort of social disaster has been shown to leave physical and psychological damage on the population (3, 4). Many suffer from depression (5), shock, or even severe heart attacks. In fact, epidemiological research has examined the influence of stressful social disaster incidents on acute myocardial infarction (AMI) such as the death of a beloved (6), earthquakes (7), war (8), and World Cup football (9).

Recent developments among big data analysis platforms have led to advanced forms of analysis methods such as Natural Language Processing (NLP) and data mining in medical fields (10). These methods can now be used to analyze consumers' responses to products or brands, predict elections via Twitter (11), predict stock sentiment momentum (12), create decision support systems (13), and predict the success of new movies (14).

Recent trends in research by Hussein et al. (15) proposed a novel semi-supervised learning model based on the combined use of random projection scaling as part of a vector space model and support vector machines to perform reasoning on a knowledge base. Additionally, Zhao et al. (16) attempted an unsupervised three-component framework to expand some pseudo contexts from web to help disambiguate a collocation's polarity. Lastly, Akhtar et al. developed a stacked ensemble method for predicting the degree of intensity for emotion and sentiment by combining the outputs obtained from several deep learning and classical feature-based models using a multi-layer perceptron network (17).

However, no research has been conducted at the domestic level thus far to examine the influence of the Sewol Ferry Disaster on social stress using sentiment analysis, despite the disaster's role as a critical domestic issue. Some studies have made minor attempts, using Twitter alone to analyze social media regarding the disaster (4), but the amount of data was not sufficient to deem it as examining effects at the domestic level. Other social science researches focused on the cause and effects of certain factors in a set timeline (18, 19).

This study analyzed the relationship between the Sewol Ferry Disaster as a social disaster and social stress by conducting sentiment analysis on data from social media. Sentiment analysis, which is an advanced form of NLP and text mining, and the analyses in this study examined data from the top social media platforms used worldwide (YouTube, Twitter, and Facebook). This study provides basic research results for the analysis of the correlation between social disasters and social stress.

## Methods

### Study Design

**Figure 1:**
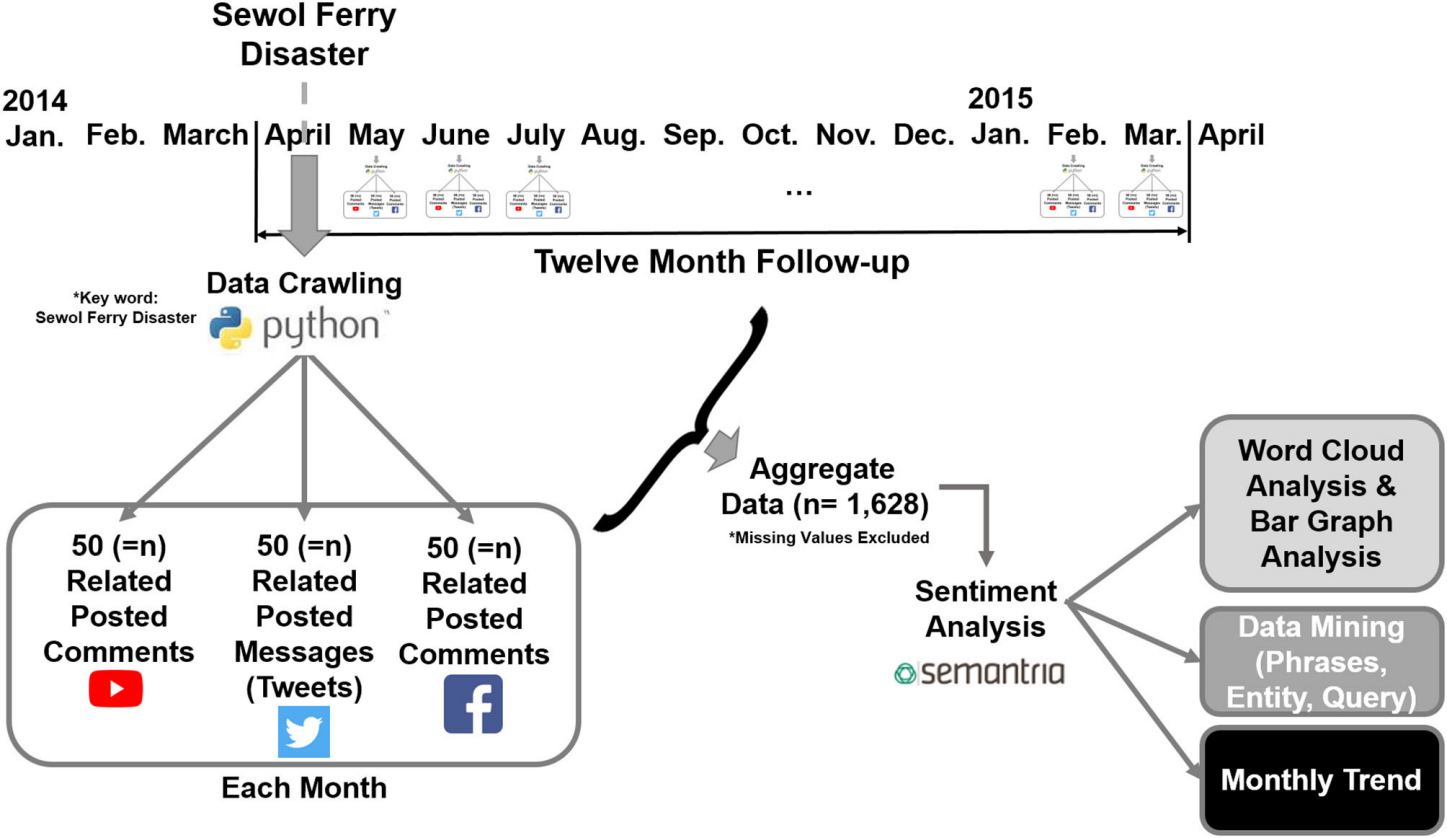
Study design of the proposed research.

The sentiment analysis study design of this article is shown in Figure 1. Centered on the keyword “Sewol Ferry Disaster,” 50 related posted comments, messages, or tweets were collected for each month. Twelve-month data were aggregated and input to the sentiment analysis machine learning algorithm of Semantria Lexalytics. Analysis of our parameter modified algorithm resulted in word cloud analysis, bar graph analysis, data mining phrases, entity, query topics, and monthly trend analysis.

**Figure 2:**
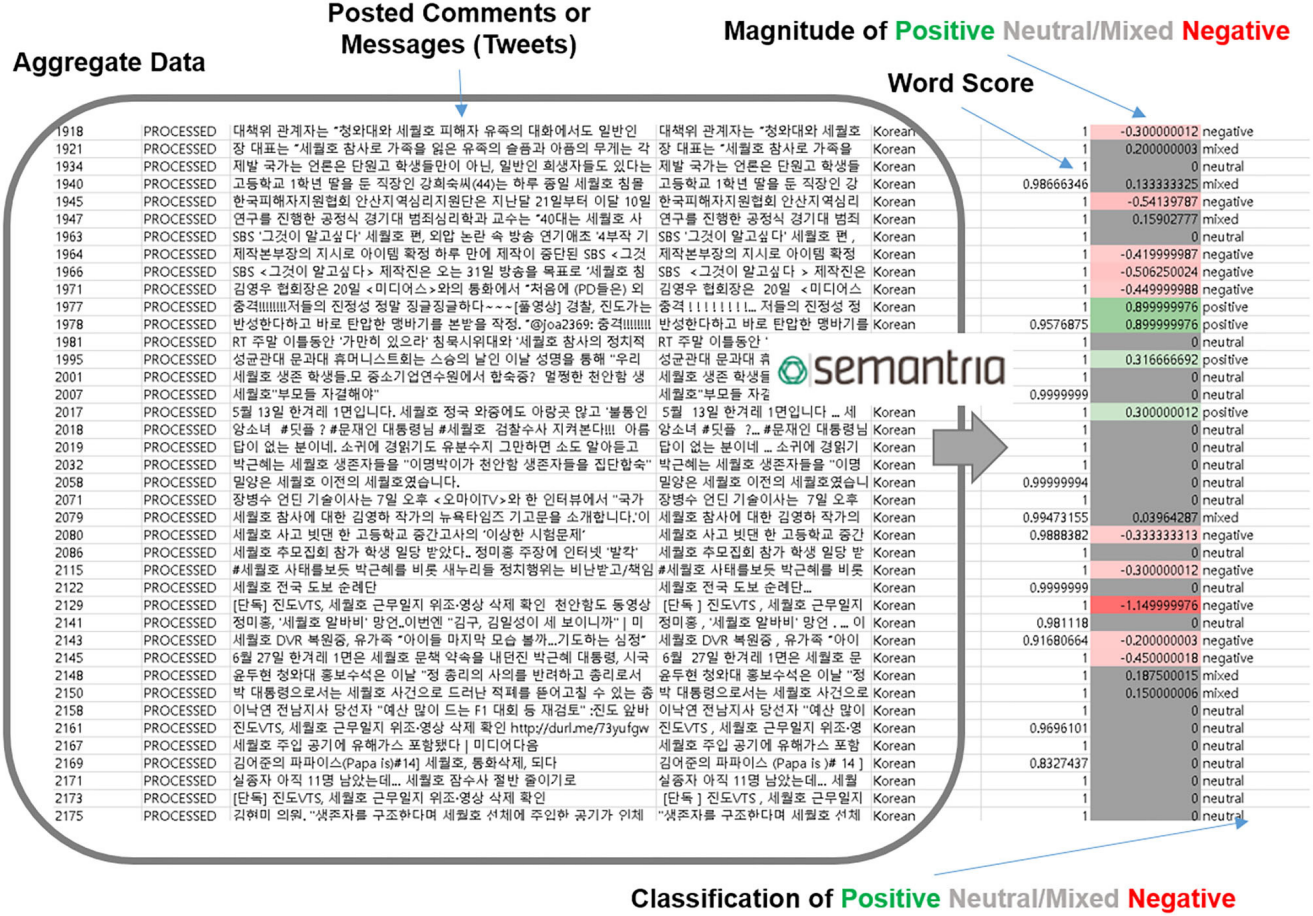
Semantria analyzing sentiment on raw aggregate data.

In this article, “phrases” refers to a small group of words that form a unit, either on their own or within a sentence in Korean. The term “entities” is used to describe a larger form of phrases, such as a sentence. The term “query topic” follows the definition provided by the Semantria Lexalytics Korean Pack, which categorizes queries into the following categories: health, science, education, culture, disaster, politics, and religion. “Positive” terms were colored green; “Neutral,” gray; and “Negative,” red. For example, Figure 2 shows a screenshot of how Semantria Lexalytics induce entity analysis from raw aggregate data.

Word score refers to the reliability of the sentiment results (from zero score to one). For example, if a certain data set's sentiment was −0.33 negative, but if the word score was 0.8, the analysis result is considered less credible compared to sentiments with 1.0 score results. Another example, “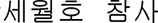 (Sewol Ferry Disaster in Korean)” was naturally categorized as “negative” sentiment, because it accompanies negative emotions such as fear, anger, and depression amongst the population. This was the same for “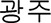 (Gwangju in Korean)” at that time, because 18th of May 1980 (~1 month after the Ferry Disaster) marked the historical Gwungju Democratization Struggle. This was related to the “anger” sentiment of the population, because ex-president Park was related to the president back in 1980 (as daughter and father), who brutally suppressed uprising Gwangju citizens at that time.

### Dataset Collection

Social media data extracted from YouTube, Twitter, and Facebook were used (Supplementary Material), as these platforms were the top-three ranked forms of media worldwide. The population was users who were randomly selected from the entire population of the aforementioned social media platforms who had posted texts related to the Sewol Ferry Disaster from April 2014 to March 2015 (12 months).

April 2014 was set as the starting date because the Sewol Ferry Disaster took place on April 16, 2014. The total number of data sets selected from YouTube, Twitter, and Facebook were 1,628: Equal sets of data that randomly selected in each month to compose a sufficient data set totaling 1,628 (refer to Figure 1). The definition of data sets refers to a composition of more than two words that form a sentence or more. All personal identifiers were deleted due to privacy issues. In the case of Facebook, <50 posts were selected for May, June, July, September, and October 2014 due to a lack of posts online. This was the same case for YouTube in January 2015.

### Sentiment Analysis and Applied AI Algorithm

Social data was extracted (data crawling) by using Python for all three platforms (20). Semantria Lexalytics (21) was used for sentiment analysis (data mining). The Semantria Application Programming Interface (API) is a cloud-based text analytics and sentiment analysis service based on advanced machine learning and natural language processing. It performs multilevel analyses of sentences incorporating parts of speech, assignment of a sentiment score from dictionaries, application of intensifiers, and determination of the final sentiment score based on machine learning techniques (22). Mostly used for business purposes, it is a tool that has recently seen increasing use in research for successful data mining (22). This tool provides an API for Excel and conducts its machine learning algorithm based on theme, entity, and category.

**Figure 3:**
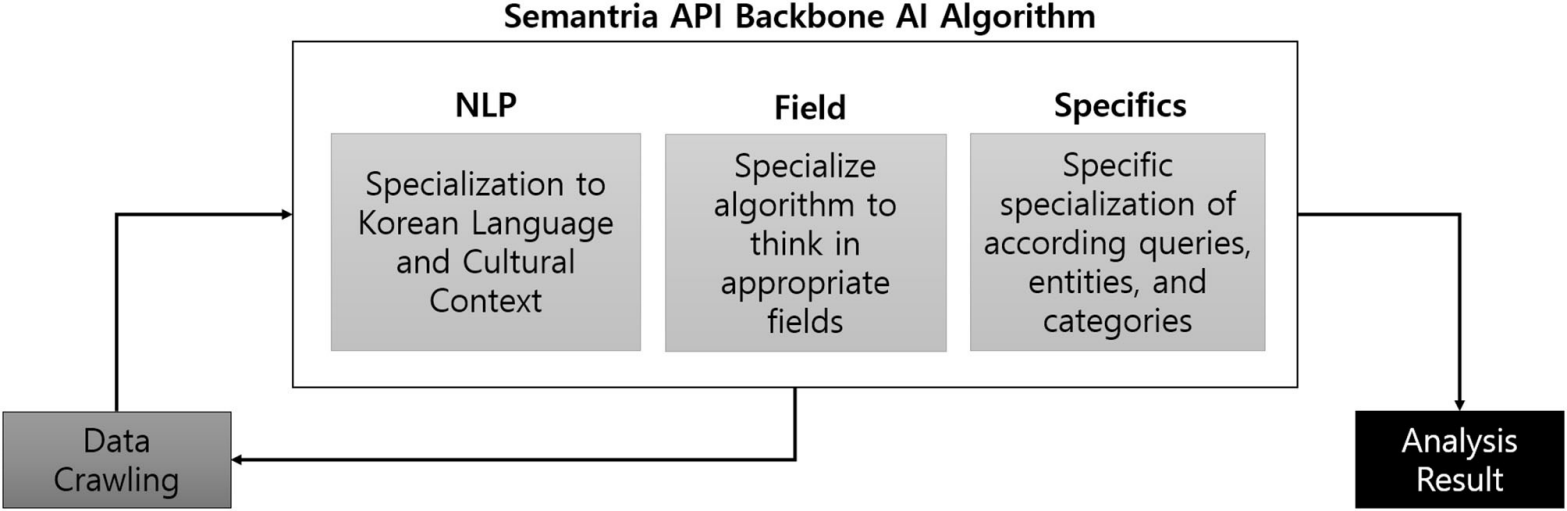
Result of modified machine learning algorithm structure.

Modification of sentiment analysis processing AI algorithms' key parameters was also conducted using Python (Figure 1). Main machine learning algorithm provided by Semantria API was used as backbone in our research, and key parameters of the AI was modified and properly trained (specialized) to properly analyze Sewol Ferry Disaster in context. Specific modification and specialization of key parameters are explained in Figure 3.

Key parameters of the algorithm were modified in mainly three terms: in terms of NLP, field, and specifics. First, parameters were specialized for the AI to properly understand Korean language in the right grammar and cultural context. Second, algorithm was modified so it may only think in appropriately set fields. For instance, some of the main fields that were set in the algorithm were “Politics,” “Disaster,” “Health,” and “Population culture.” On the contrary, fields such as “Religion,” “Technology,” or “Business” were unrelated, so these inappropriate fields were set as exclusion criteria. Lastly, queries, entities, and categories were specified, because otherwise, the AI would not know the meaning of “ex-President Park” or “Saenuri Party.” Overall settings resulted in the final “brain” of the AI to conduct proper analysis.

Analysis is divided into three sections: (a) YouTube, (b) Twitter, and (c) Facebook. Each of these three sections was then divided into two sub-sections: (a) bar graph and word cloud analysis, and (b) data mining results by phrases, entity, and query topic. In Lexalytics, phrases are defined as bigrams and noun phrases (23). Entities are the who (and some of the what) of text analytics. An entity in text is most commonly a proper noun such as a person, place, or product (https://www.lexalytics.com/technology/entity-extraction). Query topics are rules that decide whether a document belongs in a given category by looking for the presence or absence of key words and phrases (24).

### Statistical Analysis

ANOVA was used for statistical comparisons between negative, neutral, and positive sentiment under a 95% confidence level. Age, sex, and political orientation factors were adjusted. A comparison of word score groups was conducted between positive, neutral, and negative word groups. IBM SPSS 21.0 was used for ANOVA statistical analysis. All numbers were rounded up to the first decimal place.

## Results

### Bar Graph and Word Cloud Analysis

**Figure 4:**
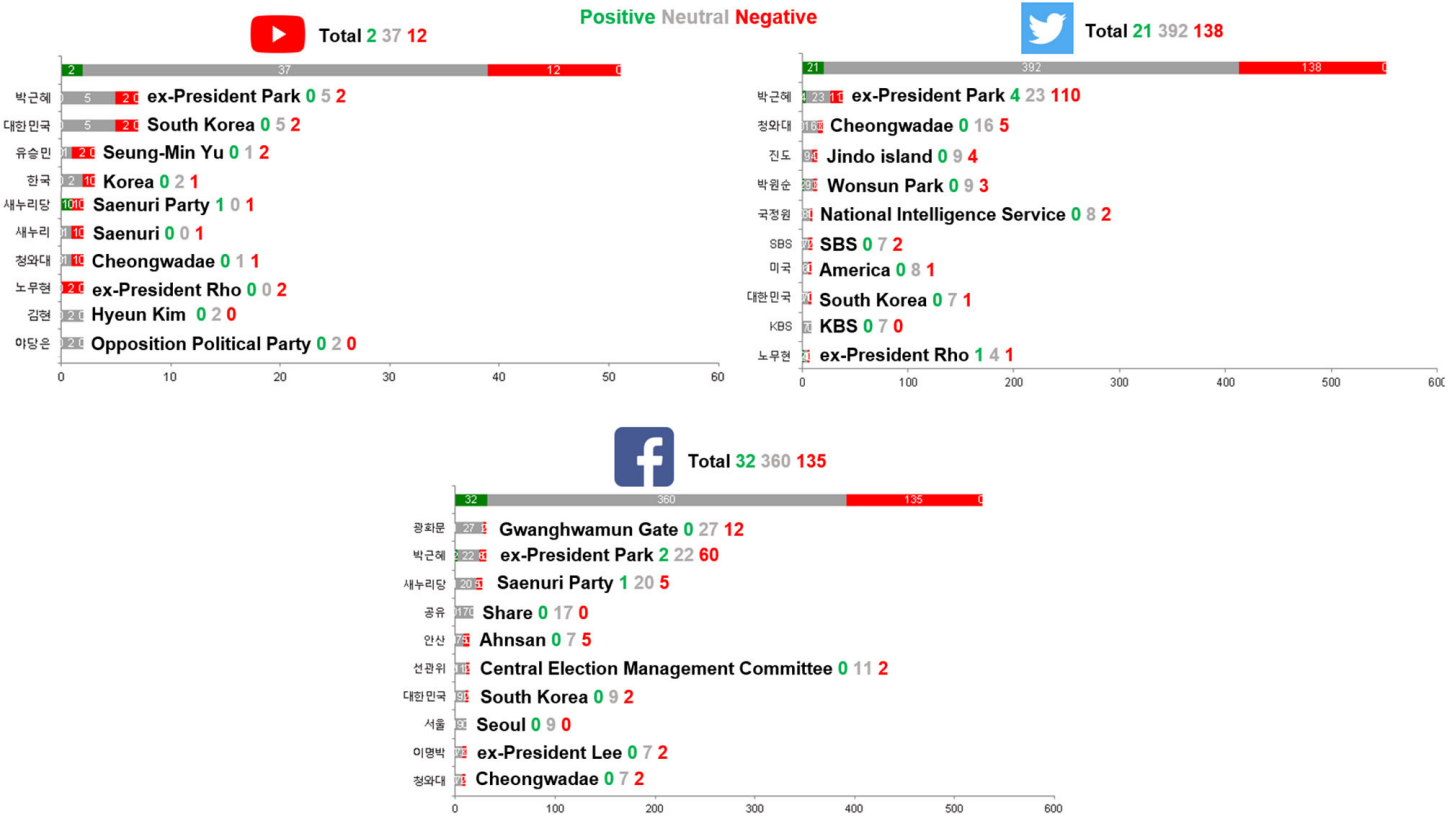
Bar graph analysis results (YouTube, Twitter, and Facebook).

Bar graph and word cloud analyses are shown in this section (Figure 4). The goal of the bar graph analysis is to show the overall sentiment state, whereas the main objective of the word cloud analysis is to show the impact of the most influential words chosen. In the bar graph analysis, the x-axis indicates the number of designated words shown, while the y-axis shows a list of the most impactful words ranked top-to-bottom (top 10 ranked words).

In YouTube, there were more negative opinions (*n* = 12) than positive opinions (*n* = 2) regarding the Sewol Ferry Disaster. In the bar graph analysis for Twitter, there were more negative opinions (*n* = 138) than positive opinions (*n* = 21) regarding the matter of the Sewol Ferry Disaster. Bar graph analysis for Facebook also showed much more negative opinions (*n* = 135) than positive opinions (*n* = 32) regarding the Sewol Ferry Disaster. Most top ranked words were targeted against the ex-president Geun-Hye Park and her policies.

**Figure 5:**
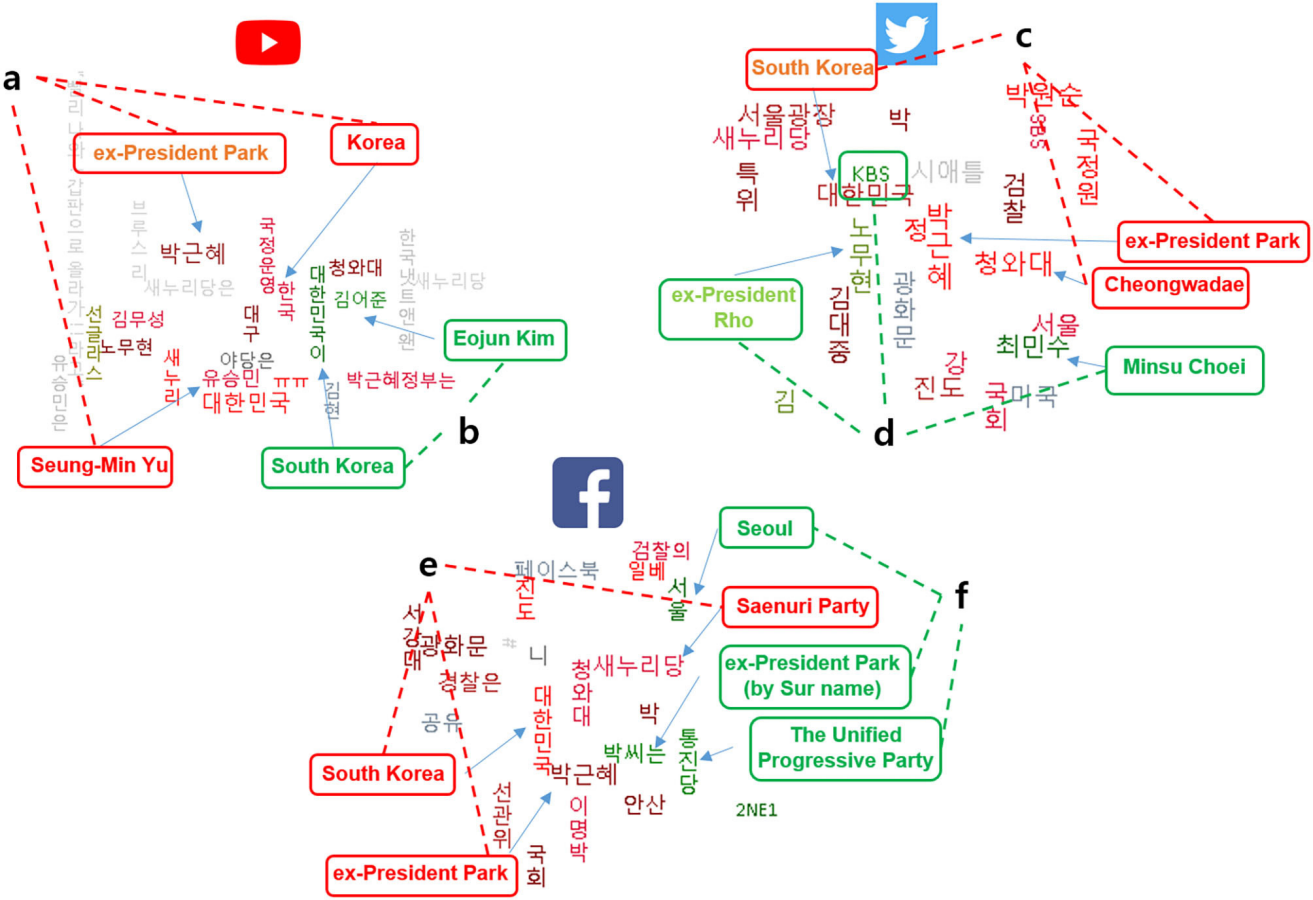
Word cloud analysis results for YouTube (a - negative, b - positive), Twitter (c - negative, d - positive), and Facebook (e - negative, f - positive).

Word cloud analysis results are shown in Figure 5. The larger the word, the larger its impact. The brighter the red of the word, the more negative weight the word represents. Also, words that are more centered meant that they were more significant and were more frequently used.

In YouTube, words such as ex-president Geun-Hye Park, South Korea, and Seung-Min Yu (a friendly South Korean politician to the ex-president) were the top three negative sentiment words in the word cloud (Figure 5a). Word cloud analysis results from Twitter showed words such as ex-president Geun-Hye Park, South Korea, and Cheongwadae (the Blue House, the Korean presidential residence) to be the top three most negative words chosen (Figure 5c). As for Facebook, the word cloud analysis results showed words such as ex-president Geun-Hye Park, South Korea, and Saenuri Party (the ex-president's political party at the time) as the top three negative words chosen (Figure 5e).

In YouTube, words such as South Korea, and Eojun Kim (Korean celebrity) were two scarcely selected positive sentiment words in the word cloud (Figure 5b). Word cloud analysis results from Twitter showed words such as KBS (Korea Broadcasting System), ex-President Mu-Hyeon, Rho, and Min-su, Choi (Korean celebrity) to be the three positive words chosen (Figure 5d). As for Facebook, the word cloud analysis results showed words such as ex-president Geun-Hye Park (by surname), Seoul, and the Unified Progressive Party (minority political party with positive impression at the time) as the three positive words chosen (Figure 5f).

### Data Mining Results by Phrases, Entity, and Query Topic

Data mining results by phrases, entity, and query topic are shown in Table 1. For YouTube, phrases with negative sentiments (*n* = 200) were more common than those with positive sentiments (*n* = 115) by 63.5%. Regarding entities, negative sentiment (*n* = 43) was larger than positive sentiment (*n* = 10) by 81.1%. Lastly, in the case of queries, negative sentiment (*n* = 9) was larger than positive sentiment (*n* = 3) by 75.0%. All difference results between negative, neutral, and positive identified with ANOVA were statistically significant at under a 95% confidence interval (*p* < 0.001).

**Table 1:** Data mining results by phrases, entity, and query topic.

**Media**	**Classification**	**Negative**	**Neutral**	**Positive**	***p*-value**	**Sentiment (percentage)**
YouTube	Phrases	200	87	115	*p* < 0.001	Negative (63.5%)
	Entity	43	140	10	*p* < 0.001	Negative (81.1%)
	Query Topic	9	26	3	*p* < 0.001	Negative (75.0%)
Twitter	Phrases	390	200	172	*p* < 0.001	Negative (69.4%)
	Entity	290	1,020	125	*p* < 0.001	Negative (69.9%)
	Query Topic	35	109	6	*p* < 0.001	Negative (85.4%)
Facebook	Phrases	362	215	253	*p* < 0.001	Negative (58.9%)
	Entity	260	1,008	82	*p* < 0.001	Negative (76.0%)
	Query Topic	24	105	8	*p* < 0.001	Negative (75.0%)

*Sentiment cases = n*.

In Twitter's case for phrases, negative sentiment (*n* = 390) was larger than positive sentiment (*n* = 172) by 69.4%. In the case of entities, negative sentiment (*n* = 290) was larger than positive sentiment (*n* = 125) by 69.9%. Lastly, regarding queries also, negative sentiment (*n* = 35) was larger than positive sentiment (*n* = 6) by 85.4%. All difference results between negative, neutral, and positive shown with ANOVA were statistically significant at under a 95% confidence interval (*p* < 0.001).

Facebook data mining phrases results showed negative sentiment (*n* = 362) to be larger than positive sentiment (*n* = 253) by 58.9%. Regarding entities, negative sentiment (*n* = 260) was again larger than positive sentiment (*n* = 82) by 76.0%. Lastly, in the case of queries, negative sentiment (*n* = 24) was larger than positive sentiment (*n* = 8) by 75.0%.

### Sentiment Analysis by Monthly Trends

**Figure 6:**
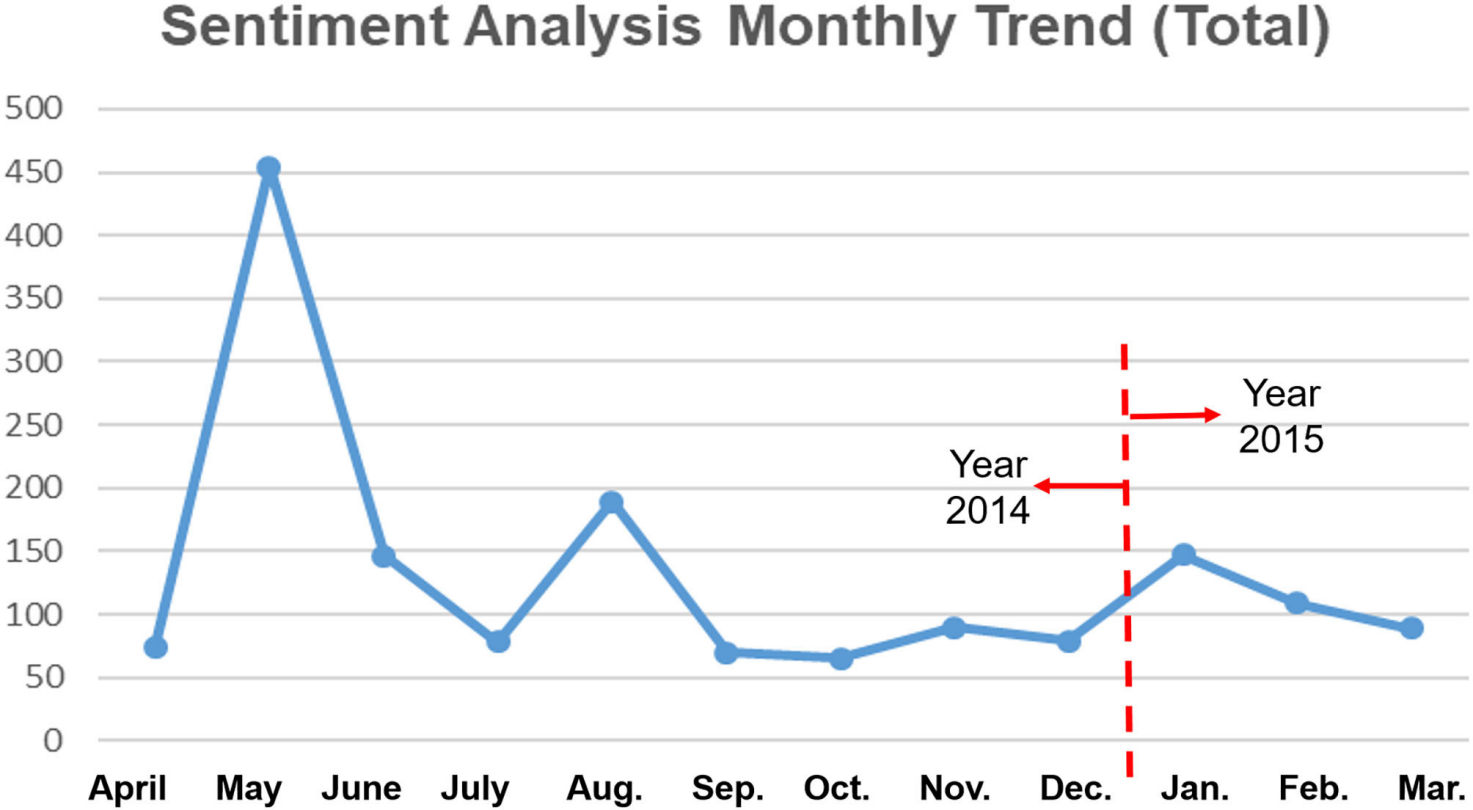
Sentiment analysis monthly trends.

A sentiment analysis by monthly trends of all social media (YouTube, Twitter, and Facebook) used in this study is shown in Figure 6. Negative sentiment boomed during the time immediately after the Sewol Ferry Disaster and then faltered as time went by. There was a momentary peak of negative sentiment in August. Negative sentiment also started to rise slightly during the period approaching the anniversary of the Sewol Ferry Disaster.

## Discussion

This research implemented machine learning-based data crawling and mining methods for sentiment analysis of the Sewol Ferry Disaster and social stress. Overall, the research results showed an undisputable negative sentiment regarding the social issue of the Sewol Ferry Disaster. Fundamental agents that led to negative sentiments were ex-president Geun-Hye Park and her related political parties (Saenuri Party) and politicians (Seung-Min Yu).

Some broadcasting systems or celebrities were positively mentioned (KBS, Eo-jun, Kim, Min-su, Choei, or 2NE1) but were less significant or less frequently used. KBS at the time was aggressive and critical toward the government's incompetent reaction to the disaster. This seems to have gained some positive view and support from the citizens. Also, ex-president Rho was positively mentioned in contrast to president Park at the time. This was because ex-president Rho stood for a symbol of peace for the citizens due to his accomplishment of being selected as the candidate of the Nobel Peace Prize in 2007. Actors and celebrities such as Eo-jun Kim, Min-su Choei, and 2NE1 gained positive view for providing empowerment and entertainment in depressive time. “South Korea” and “ex-President Park” may have been mentioned negative and at the same time positive by some people but were less significant and less frequently used positive (words far away from the center indicate less significance) than they were used negative.

Overall as time passed, the negative sentiment among the population waxed and waned due to the sustained social stress caused by the government's insufficient measures to tackle this issue. The distinct peak seen during August seemed to be due to the following issues. On the 19th of August in 2014, the family members of students who died in the Sewol Ferry Disaster protested against the “Sewol Ferry Special Law” passed by the government because the law did not provide sufficient compensation. In addition, Sewol Ferry Disaster-related investigation disputes were fierce during that month.

In January 2015, nearing the anniversary of the Sewol Ferry Disaster, “Sewol Ferry Disaster d-Day +300 Press Conference” took place. In addition, the Sewol Ferry ship salvage issue added to the triggering of negative memories of the Sewol Ferry Disaster among the devastated population.

This research provided evidence of the disaster's negative psychological impact on the Korean population. The results of this study are especially meaningful considering that the ex-president Geun-Hye Park was eventually impeached by the citizens of South Korea in December 2016 (implemented in March of 2017). Her impeachment was a result of several factors, but the consistent negative sentiment centered on the ex-president over a long period of time may have influenced the events.

This research is limited by the core mechanism of Twitter, which mainly selects samples that are the most-recognized among the data (25); our raw data were chosen from this pool. Although the mechanisms of YouTube and Facebook are not clearly stated, considering that the functions of social media are typically similar, this may have caused some bias in the research results.

Social media usage is ever increasing; this is expected to result in the production of even more social data, which will be useful for future research that uses sentiment analysis (26). In addition, learning models from computational biology and medicine have been successfully applied in other medical fields in recent research (27, 28). The significance of this research is that it is the first study to conduct NLP-based sentiment analysis on data from the three largest social media platforms (YouTube, Twitter, and Facebook) regarding the issue of the Sewol Ferry Disaster. Future research should examine whether the negative sentiment identified in this study had any influence on the incidence rate of depression or cardiovascular diseases such as heart attack and acute stroke.

## Data Availability Statement

All datasets generated for this study are included in the article/supplementary material.

## Author Contributions

M-JL constructed the main idea of this article, and conducted NLP-based sentiment core analysis. S-JL contributed significantly in writing the article. T-RL and J-SJ provided critical professional advice and modifications to the article. EK supervised and managed the entire projected. All authors contributed to the article and approved the submitted version.

## Conflict of Interest

The authors declare that the research was conducted in the absence of any commercial or financial relationships that could be construed as a potential conflict of interest.
